# Phosphorus and Defoliation Interact and Improve the Growth and Composition of the Plant Community and Soil Properties in an Alpine Pasture of Qinghai-Tibet Plateau

**DOI:** 10.1371/journal.pone.0141701

**Published:** 2015-10-29

**Authors:** Juan Qi, Zhongnan Nie, Ting Jiao, Degang Zhang

**Affiliations:** 1 College of Grassland Science, Gansu Agricultural University, Lanzhou, Gansu, People’s Republic of China; 2 Department of Economic Development, Jobs, Transport and Resources, Hamilton, Victoria, Australia; Chinese Academy of Sciences, CHINA

## Abstract

Pasture degradation caused by overgrazing and inappropriate fertiliser management is a major production and environmental threat in Qinghai-Tibet Plateau. Previous research has focused on the effects of mixed nitrogen (N) and phosphorus (P) fertiliser and reduced grazing pressure on the plant community of the grassland; however, the role of P and how it interacts with various defoliation (the process of the complete or partial removal of the above-ground parts of plants by grazing or cutting) intensities on the plant and soil of the grassland ecosystem have not been quantified. A field experiment was conducted to quantify how P application in combination of defoliation pressure could impact the dynamic change of the plant and soil in a native alpine grassland ecosystem of the Qinghai-Tibet Plateau, China, from May 2012 to September 2014. A split-plot design with 4 replicates and repeated measures was used to determine the growth and composition of plant community and soil physical and chemical properties under various levels of P fertiliser and defoliation intensity. The results showed that applying 20 kg P/ha increased the herbage yield of *Melissitus ruthenica* by 68% and total pasture yield by 25%. Close defoliation favoured the growth and plant frequency of the shorter species, whereas lax defoliation favoured that of the taller plant species. Medium P rate and cutting to 3 cm above ground gave an overall best outcome in pasture yield, quality and frequency and soil moisture and nutrient concentration. Application of P fertiliser with a moderate defoliation pressure to promote legume growth and N fixation has the potential to achieve multiple benefits in increasing pasture and livestock production and improving environmental sustainability in the alpine pasture of Qinghai-Tibet Plateau, a fragile and P-deficient ecosystem zone in China and its western neighbouring countries.

## Introduction

The Qinghai-Tibet Plateau is the youngest, highest and largest plateau on the earth with an area of about 2.57 million (m) km^2^, representing over 26% of the total land area in China [1]. With an average altitude of about 4,500 m above sea level across the plateau, the temperature can be as low as -40°C in winter, and the annual growth period of pastures is only about 90 days. The annual precipitation of the plateau is also low; however, the water resource is rich with many rivers, lakes, glaciers and wetlands distributed throughout the region, accounting for about 20% of the total water resource in China [2].

The area of pastures in Qinghai-Tibet Plateau is approximately 150 m ha, 62.4% of the national total of China. Largely due to mismanagement, particularly overgrazing, pasture degradation has become a major problem that has a significant impact on the productivity of the grasslands as well as environmental sustainability [3]. The degraded pasture area was estimated to be over 30% of the total grassland area available for grazing in the plateau, which has led to extensive soil erosion, dust storms and water pollution to rivers and waterways [4]. In Gannan Tibetan Prefecture of Gansu Province, for instance, the groundcover by pastures has decreased by 35% and pasture yield decreased by 15% since the 1980s [5].

Grazing management is a key element of the grassland ecosystems to produce food and fibre from livestock for human beings [6]. Appropriate grazing pressure on a particular pasture depends on a wide range of conditions such as the climate, pasture species and the soil, and it is critical to understand how plant growth is impacted by grazing at given soil type/fertility and climatic conditions before sound grazing management guidelines can be developed. Studies [7–9] have found that inappropriate grazing management, particularly overgrazing, could alter the competition between the species of the pasture community, leading to increased population of weeds and toxic plants and pasture degradation. Overgrazing and its adverse impact on plant cover and soil induced serious soil erosion in the rangeland of the northern Tibet Plateau [3]. Fencing to exclude/control grazing has increased pasture productivity and improved the forage grass functional groups in a *Kobresia*-dominated meadow in the Qinghai-Tibet Plateau [9].

There is a general consensus that grazing can increase species richness whereas fertiliser application can increase pasture production but reduce species richness [10–13]. The type of nutrients may play an important role in species richness and Phosphorus (P) limitation appears to favour a higher species richness than N limitation for low productive plant communities [14]. Yang et al. reported that, while grazing can influence the growth and competition between pasture species, fertiliser application played a more important role in altering pasture yield, botanical composition and biodiversity in Qinghai-Tibet Plateau [15]. P is a key element for plant growth and about 18 Tg of P per annum is currently used globally as fertiliser [16]. On soils that are P-deficient and consequently cannot support optimum production, P fertiliser and/or manure is often applied at rates that purposefully exceed the rates of P removal and loss, so that P accumulates in the soil [17]. Reports [9,18,19] have shown that available P concentrations in the pasture soil of Qinghai-Tibet Plateau is often very low (<6 mg/kg soil). However, little is known of the effects of P fertiliser on individual species of the ‘native’ grasslands in these environments. Fertiliser studies have concentrated on mixing P with nitrogen (N) and other nutrients, and/or with other management treatments, leading to mixed messages on whether P could play a role in these harsh environments as it does in more favourable environmental conditions. For instance, a study by Yang et al. revealed that sedges and legumes declined with increasing rates of fertiliser application and disappeared in year 3 after 1200 kg/ha of Diammonium Phosphate (DAP) was applied in an alpine pasture of eastern Qinghai-Tibet Plateau [15]. It is unclear whether this detrimental effects to legumes and sedge were due to the N and P applied or their interactions, or competitions between these species and grasses since many studies [20,21] have found that P could significantly increase the growth of pasture legumes and is required at higher concentrations for legumes to grow and compete with grasses.

With enormous pressure from local and central Chinese governments to halt grassland degradation and improve environmental health and sustainability, and increasing demand for food and fibre from the grazing systems by the local residents [22], it is imperative to develop appropriate pasture management guidelines for various regions of the Qinghai-Tibet Plateau. Grazing and fertiliser management is the core of grassland management systems; however, there has been little information on how defoliation (the process of the complete or partial removal of the above-ground parts of plants by grazing or cutting), P fertiliser and their interactions act on pasture growth/competition, plant nutrient levels and soil physical and chemical properties in this fragile environment of Qinghai-Tibet Plateau [23]. This information is critical for developing grassland management strategies and policies.

The objectives of the study were 1) to investigate the impact of defoliation intensity, P fertiliser and their interactions on the herbage production, botanical composition and frequency of major pasture species/categories of an alpine meadow in Qinghai-Tibet Plateau; 2) to examine how these factors could affect the soil moisture and nutrients, and nutrients of the pasture through the above- and below-ground plant growth and competition; and 3) to develop defoliation and fertiliser management strategies/recommendations that could be incorporated in guidelines and regulations to improve the productivity and sustainability of alpine meadow for the local producers, and the local and central governments.

## Materials and Methods

### Study site

The experiment was conducted in a native alpine pasture of the Qinghai-Tibet Plateau at Tianzhu Alpine Grassland Experimental Station, Gansu Agricultural University, Tianzhu County, Gansu Province, China from May 2012 to September 2014. Tianzhu (36°31′-37°55′N, 102°07′-103°46′E) is located at the eastern end of Qilian Mountain with an altitude of 2900–4300 m a.s.l. The long-term mean annual air temperature was 0.8°C with monthly mean temperature being 12.4°C in July (highest in the year) and -18.3°C in January (lowest in the year). July and August were the only months with maximum daily air temperature > 10°C and frost-free period of 30 days; therefore, the growing season was very short although some of the native plants can grow up to 130 days/year. The long-term mean annual rainfall was 416 mm with most of the rain falling from July to September. The mean annual evaporation was 1430 mm, 3.4 times of the annual rainfall.

The pasture species in the region were diverse, and the major species included grasses (predominantly *Elymus nutans*), legumes (predorminantly *Melissitus ruthenica*), sedge (*Kobresia humilis*) and forbs (e.g. *Potentilla bifurca*, *Gentiana straminea*). The soil was mountain black soil (chernozem) with pH = 7.5 and organic matter of 12% in the 0–40 cm soil profile.

### Experimental design and treatments

Two factors were examined in this study: defoliation [24] intensity and P application. Defoliation intensity included 3 levels: 1) cut to 1 cm above ground; 2) cut to 3 cm above ground; and 3) cut to 5 cm above ground. P application also included 3 levels: 1) low P—no fertiliser was applied; 2) medium P– 20 kg P/ha of dibasic sodium phosphate (DSP; Na_2_HPO_4_) was applied in May–June annually; and 3) high P– 40 kg P/ha of DSP was applied at the same time as for level 2. The DSP product was made from rock phosphate and supplied by Damao Chemical Company, Tianjin, China. The DSP was weighed and applied evenly to individual plots based on their designed P application rates.

The 3 cutting intensity levels combined with the 3 P application levels made up 9 factorial treatments. A split-plot design was used with cutting intensity as the main plot and P application as the sub-plot replicated for 4 times. The sub-plot size was 1 m x 1 m = 1 m^2^ with a 50 cm gap (buffer area) between the sub-plots and between replicates. Small plot size was used to ensure uniformity in species composition and soil properties in this variable and diverse grassland.

### Measurements

#### Herbage yield and botanical composition

Herbage yield was measured by harvesting individual treatment plots to their designed heights each year from 2012 to 2014. Subsamples were then taken from the herbage samples from each plot, freshly weighed and oven dried at 100°C for 24 hours to determine the herbage yield. An additional subsample was also taken from the fresh herbage sample of individual plots and dissected into *E*. *nutans*, *K*. *humilis*, *M*. *ruthenica*, forbs and dead matter. The component samples were oven dried at 100°C for 24 hours to calculate botanical composition (the percentage of the herbage mass of one species or category to the total herbage mass on a dry matter basis) and herbage yield of individual species and sward component [24; 25].

#### Plant height and frequency

Plant height was measured by recording the height of 10 randomly selected plants of *Elymus nutans*, *Kobresia humilis* and *M*. *ruthenica* within a plot before each harvest in 2013 and 2014. The height was recorded when the thumb of an observer moved down along a ruler and touched the plant of selected species [25]. The 10 readings were averaged to give the means of individual treatment plots. At the same time for measuring plant height, plant frequency was measured using a 0.5 x 0.5 m^2^ quadrat subdivided into 10 cm by 10 cm grids. The quadrat was randomly placed in each plot and plants of *E*. *nutans*, *K*. *humilis*, *M*. *ruthenica* and other species that touched a pin underneath the crossing of grids were recorded. Plant frequency was calculated as F = R/25*100 while F is plant frequency (%) and R the number of touched plants from each quadrat.

#### Herbage phosphorus, crude protein and acid detergent fibre content

The concentration of P, crude protein and acid detergent fibre (ADF) in the herbage was measured using the herbage samples harvested on 30 August 2013. Subsamples of *E*. *nutans*, *K*. *humilis*, *M*. *ruthenica*, forbs and dead matter from the herbage samples were oven dried at 65°C for 48 hr before being ground. For P concentration, 0.5 g of the ground herbage from each sample was added in a flask, mixed with 5 ml of H_2_SO_4_ and HNO_3_ solution (H_2_SO_4_: HNO_3_ = 2.5: 1), then shaken until they were fully mixed. The solution was heated over a low flame until brown smoke appeared. The solution was cooled and solution of HNO_3_ and HCLO_4_ (HNO_3_: HCLO_4_ = 1: 1) was added. The solution was heated again until it became clear and was topped up to 50 mL with ammonium-free distilled water. Five mL of the solution was taken and put to a 25 mL flask. Two drops of phenolphthalein were added followed by drops of 4% NaOH until the solution turned reddish. Solution of 2% H2SO4 was then added until the red color faded. A solution of ammonium molybdate and sodium sulfite (2 mL) and hydroquinol (1 mL) was added, topped up with distilled water to 25 mL. The solution was allowed for color development for 30 min before colorimetric assay at 660 nm was used to determine the P concentration using standard P solutions for comparison.

For crude protein concentration, N was determined by a small-scale Kjeldahl digestion [26]. One gram of the ground herbage from each sample was added in a flask, mixed with 10 ml of H_2_SO_4_ and 3 g of CuSO_4_·5H_2_O and K_2_SO_4_ (CuSO_4_·5H_2_O: K_2_SO_4_ = 1: 10) and shaken until they were thoroughly mixed. The solution was heated from a low to high flame until the solution became clear. The solution was then topped up to 100 mL with ammonium-free distilled water, and 10 mL of the solution was mixed with 4 mL 40% NaOH and distilled using a Kjeldahl apparatus for 4 min. The released NH_3_ through distillation was absorbed by 1% boric acid, and then titrated against 0.02 mol L^-1^ HCl to determine the N content in the sample. The N concentration was multiplied by 6.25 to calculate the crude protein content.

For ADF, 1 g of the ground herbage from each sample was put in a nylon bag (400 mesh) tied with a nylon thread. The nylon bag was then put into a 500 mL beaker filled with 100 mL acidic detergent. A few drops of decahydronaphthalene was added before the sample bag was boiled for 1 h on a low flame. The nylon bag was then washed with warm water until there was no visible substances coming from the bag. The nylon bag with its residuals was soaked with a small amount of acetone for 10 min. After the acetone was volatilized completely, the nylon bag was oven dried at 80°C until constant weight to determine ADF.

#### Soil moisture and nutrients

Gravimetric soil moisture was measured by taking 3 4-cm diameter cores to a depth of 30 cm randomly from each plot on 30 August 2013. The cores were cut into 0–10, 10–20 and 20–30 cm segments and bulked across the same depth for each plot. The samples were weighed freshly and then oven dried at 100°C until constant weight was obtained. The moisture content of the soil was calculated as SM = (FW − DW)/FW*100 where SM is soil moisture content (%), FW fresh weight and DW dry weight of the sample.

Soil nutrients were measured by taking 3 4-cm diameter and 30-cm deep cores from each plot on 30 August 2013. Cores from each plot were mixed thoroughly after visible roots and stone were removed. The soil sample was divided into two parts: one was air-dried and ground to pass a 1-mm mesh for available N, P and K study, and the other was air-dried and ground to pass a 0.25-mm mesh for total soil N, P and potassium (K) determination.

As for the measurement of herbage crude protein, total soil N was determined using a small-scale Kjeldahl digestion procedure [26]. Briefly, a small amount of dried soil (passing 0.25 mm sieves) mixed with H_2_SO_4_, CuSO_4_·5H_2_O and K_2_SO_4_ was heated and then topped up with ammonium-free distilled water. The solution was mixed with 4 mL 40% NaOH and distilled using a Kjeldahl apparatus to release NH_3_ to determine N and protein content. Available soil N was extracted and determined using NaOH, H_3_BO_3_, and HCl. Total P and available P were extracted with HF—HNO_3_–HClO_4_ and sodium bicarbonate, respectively, and then determined by the molybdenum blue method. Total K and available K were extracted with HF—HNO_3_–HClO_4_ and ammonium acetate, respectively, and then determined by flame photometry.

### Statistical analysis

Data were analysed using the General Analysis of Variance (ANOVA) of GENSTAT Release 14.1 [27]. A split-plot model with repeated measures was used to analyse the effects of cutting, phosphorus and their interactions on herbage yield, botanical composition, plant height and plant frequency. A split-plot model was used to analyse the effects of cutting, phosphorus and their interactions on the concentrations of nutrients in the herbage and soil. A split-split-plot model with repeated measures was used to analyse gravimetric soil moisture at different depths. The source of variation and degree of freedom for these models are given in S1 Table. Significant difference was denoted as *P* < 0.05 or *P* < 0.01 when the probability was less than 5 or 1%, respectively. *P* < 0.1 (probability was less than 10%) was used for marginal effects between treatments, and *P* > 0.1 for no significant difference between treatments.

## Results

### Herbage yield

#### Defoliation effect

There was a significant (*P* < 0.01) difference in herbage yield of *K*. *humilis* between the defoliation treatments (Table 1). The mean herbage yield of this species was 533 kg DM/ha when pasture was cut to 1 cm above ground, 12% higher than when pasture was cut to 3 cm and 44% higher than when pasture cut to 5 cm. The total herbage yield showed a similar trend, and the effect of defoliation was marginal (*P* < 0.1). There were no significant (*P* > 0.1) differences in the herbage yield of *E*. *nutans*, *M*. *ruthenica*, forbs and dead matter between the defoliation treatments.

**Table 1:** Mean herbage yield (kg DM/ha) of major species/categories under various defoliation regimes (cut to 1, 3 and 5 cm above ground), phosphorus application (low, medium and high) and time from July 2012 to September 2014.

Treatment	*Elymus nutans*	*Kobresia humilis*	*Melissitus ruthenica*	Forbs	Dead matter	Total
*Defoliation*						
**1**	275	533	726	1156	189	2817
**3**	313	476	711	1126	163	2735
**5**	312	371	605	1120	124	2476
***s*.*e*.*m*.**	*54*.*8*	*21*.*0* **	*46*.*8*	*73*.*4*	*22*.*6*	*94*.*3* ^*†*^
*Phosphorus*						
**Low**	254	424	465	1091	131	2280
**Medium**	323	491	782	1108	174	2848
**High**	323	465	796	1203	170	2900
***s*.*e*.*m*.**	*35*.*0*	*32*.*4*	*39*.*3* **	*68*.*1*	*17*.*1*	*85*.*0* **
*Time*						
**2012**	115	353	375	1292	159	2121
**2013**	203	558	792	827	139	2519
**2014**	582	469	875	1283	178	3388
***s*.*e*.*m*.**	*31*.*4* **	*33*.*4* **	*42*.*1* **	*69*.*2* **	*14*.*9* ^*†*^	*99*.*7* **

**P<0.01;

^*†*^ *P*<0.1.

#### Phosphorus effect

There were significant (*P* < 0.01) differences in the *M*. *ruthenica* and total herbage yield between the P treatments (Table 1). The medium and high P treatments on average produced 789 kg DM/ha of *M*. *ruthenica* and 2874 kg DM/ha of total herbage, 70% and 26% higher than that under the low P treatment, respectively. Though it varied in a similar trend to *M*. *ruthenica* and total herbage yield between treatments, the herbage yield of *E*. *nutans*, *K*. *humilis*, forbs and dead matter did not differ significantly.

#### Time effect

There were significant (*P* < 0.01) differences in the herbage yield of *E*. *nutans*, *K*. *humilis*, *M*. *ruthenica*, forbs and the total between the years of harvest (Table 1). The herbage yield of *E*. *nutans*, *M*. *ruthenica* and the total were highest in 2014 and lowest in 2012. The herbage yield of *K*. *humilis* was highest, and of forbs lowest in the second year (2013). There was a marginal effect on the herbage yield of dead matter.

#### Fertiliser by defoliation interaction

**Fig 1:**
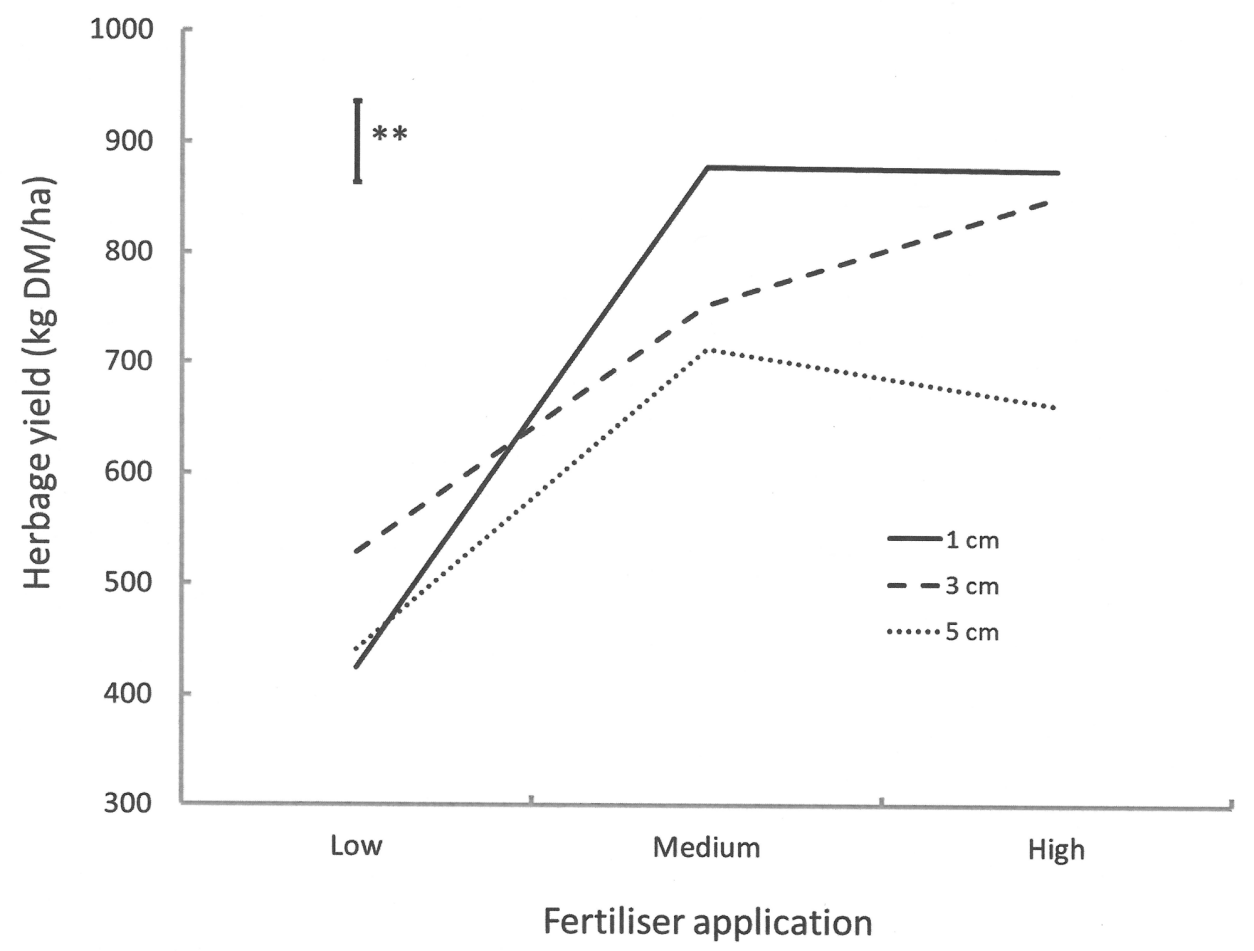
Interaction between P fertiliser and defoliation treatments on the herbage yield of *M*. *ruthenica*. Bar represents the standard error of the mean; **P < 0.01.

There was a significant interaction in the herbage yield of *M*. *ruthenica* between P and defoliation treatments (Fig 1). When pasture was cut to 3 cm above ground, the herbage yield of this legume increased with increasing P application rates. However, the herbage yield of the species did not increase further from medium to high P rate when pasture was cut either to 1 cm or 5 cm above ground.

### Botanical composition

**Fig 2:**
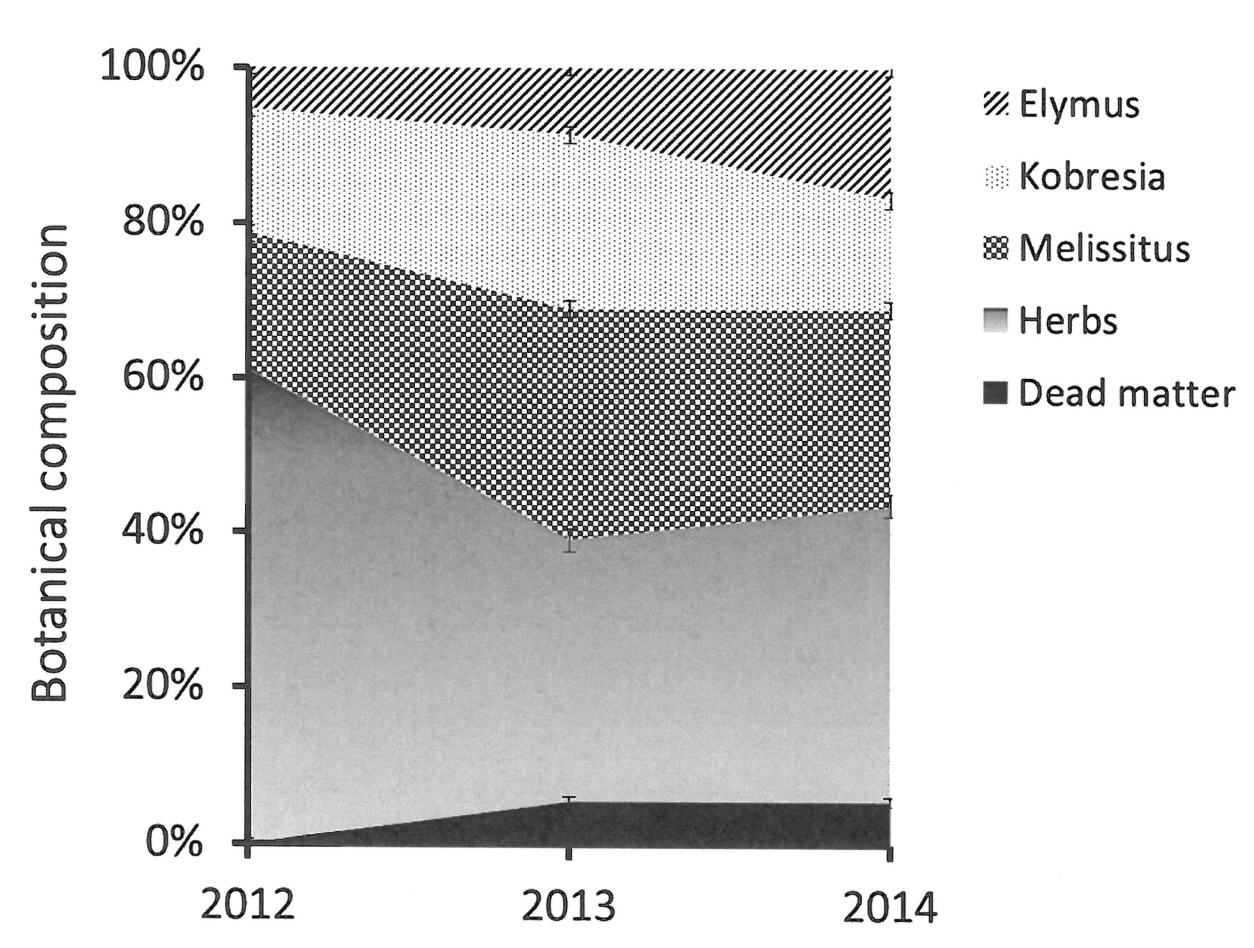
Change in botanical composition (%) of various species/categories (*Elymus nutans*, *Kobresia humilis*, *M*. *ruthenica*, forbs and dead matter) from 2012 to 2014. Bars represent the standard error of the mean.

While the botanical compositions of individual pasture components responded to the P fertiliser and defoliation treatments in a similar manner to their relevant herbage yields, their most significant (*P* < 0.001 for all components except dead matter which was not significantly different) responses were to the year of harvest (Fig 2). The botanical composition of *E*. *nutans* was about 5% in year 1 (2012) and increased to 17% after 3 years of experimental treatment. The botanical composition of *K*. *humilis*, *M*. *ruthenica* all increased from year 1 to year 2, but declined from year 2 to year 3. The forbs were the dominant species in year 1 (> 60%) and declined to < 40% in years 2 and 3.

### Plant height and frequency

#### Plant height

There were significant (*P* < 0.05) differences in plant height of *E*. *nutans* and *K*. *humilis* between P fertiliser treatments and between years (Table 2). The plant height of *E*. *nutans* and *K*. *humilis* for medium P treatment was higher than low P treatment; but there was little difference between the medium and high P treatments. The plant height of *E*. *nutans* and *K*. *humilis* was higher in 2014 than 2013. The plant height of *M*. *ruthenica* was not affected by P application and was marginally higher in 2014 than 2013.

**Table 2:** Mean plant height (cm) of major species under various fertiliser regimes (low, medium and high) and in different years (2013 and 2014).

Phosphorus	*Elymus nutans*	*Kobresia humilis*	*Melissitus ruthenica*	Year	*Elymus nutans*	*Kobresia humilis*	*Melissitus ruthenica*
**Low**	57.9	12.6	8.3	2013	49.5	11.3	9.2
**Medium**	64.6	14.8	19.1	2014	71.5	16.7	17.4
**High**	59.1	14.5	12.5	*s*.*e*.*m*.	*1*.*22* *	*0*.*63* *	*3*.*4* ^*†*^
***s*.*e*.*m*.**	*1*.*67* *	*0*.*61* *	*4*.*26*				

*P<0.05;

^*†*^ *P*<0.1.

#### Plant frequency

In contrast to plant height, there were significant (*P* < 0.05) differences in plant frequency of *M*. *ruthenica*, but not the grass and other species (forbs etc.) between the defoliation treatments (Table 3). Cutting to 3 cm above ground had a higher plant frequency than cutting to either 1 cm or 5 cm above ground for *M*. *ruthenica*. There was a marginal effect of defoliation on plant frequency of *K*. *humilis* with cutting to 1 or 3 cm being higher than 5 cm.

**Table 3:** Mean plant frequency (%) of major species/categories under various defoliation regimes (cut to 1, 3 and 5 cm above ground), phosphorus application (low, medium and high) and time (2013 and 2014).

Treatment	*Elymus nutans*	*Kobresia humilis*	*Melissitus ruthenica*	Other species
*Defoliation*				
**1**	2.0	64.3	58.6	29.0
**3**	4.7	64.0	62.9	29.6
**5**	3.3	57.5	55.9	28.1
***s*.*e*.*m*.**	*1*.*07*	*1*.*87* ^*†*^	*1*.*33* *	*2*.*37*
*Phosphorus*				
**Low**	2.7	60.8	53.2	28.5
**Medium**	2.8	60.7	63.2	29.5
**High**	4.6	64.3	61.0	28.7
***s*.*e*.*m*.**	*0*.*75*	*1*.*35*	*1*.*99* **	*1*.*64*
*Time*				
**2013**	2.4	38.0	36.2	42.5
**2014**	4.2	85.9	82.1	15.3
***s*.*e*.*m*.**	0.68^*†*^	0.86**	1.62**	1.48**

**P<0.01;

*P<0.05;

^*†*^ *P*<0.1.

There was a significant (*P* < 0.05) difference in plant frequency of *M*. *ruthenica* only among the major species in the plant community between the P fertiliser treatments (Table 3). The mean plant frequency of *M*. *ruthenica* was 63% for the medium P treatment, 19% higher than for the low P treatment.

There were significant differences in plant frequency of *K*. *humilis*, *M*. *ruthenica* and other species between 2013 and 2014 (Table 3). The mean plant frequency of *K*. *humilis* and *M*. *ruthenica* was 86 and 82% in 2014, which was twice as frequent as in the previous season. On contrary, the mean plant frequency of other species declined 64% from 2013 to 2014. The mean plant frequency of *E*. *nutans* varied marginally between years with similar trend to that of *K*. *humilis* and *M*. *ruthenica*.

### Herbage phosphorus, protein and fibre content

#### P concentration

There were significant (*P* < 0.05 or 0.01) differences in the P concentration of *E*. *nutans* and *M*. *ruthenica* between the defoliation treatment (Table 4). The P concentration of *E*. *nutans* was highest when pastures were cut to 1 cm above ground whereas that of *M*. *ruthenica* was highest when pastures were cut to 3 cm above ground. There were no significant differences in the P concentration of *K*. *humilis*, forbs, dead matter and the mean across all species.

**Table 4:** Phosphorus (P) concentration (%), crude protein (CP) content (%) and acid detergent fibre (ADF) content (%) of major species/categories under various defoliation regimes (cut to 1, 3 and 5 cm above ground) and P application (low, medium and high).

Defoliation	*Elymus nutans*	*Kobresia humilis*	*Melissitus ruthenica*	Forbs	Dead matter	Mean
*P concentration affected by defoliation*						
**1**	0.26	0.21	0.29	0.21	0.26	0.24
**3**	0.18	0.23	0.33	0.21	0.26	0.24
**5**	0.24	0.22	0.29	0.20	0.24	0.24
***s*.*e*.*m*.**	*0*.*017* *	*0*.*008*	*0*.*006* **	*0*.*010*	*0*.*040*	*0*.*004*
*P concentration affected by P application*						
**Low**	0.19	0.15	0.24	0.15	0.20	0.19
**Medium**	0.24	0.22	0.30	0.21	0.24	0.24
**High**	0.25	0.29	0.37	0.26	0.32	0.30
***s*.*e*.*m*.**	*0*.*021*	*0*.*011* **	*0*.*010* **	*0*.*011* **	*0*.*022* **	*0*.*008* *
*CP affected by defoliation*						
**1**	3.8	5.5	10.2	6.1	4.7	6.1
**3**	4.5	6.1	10.5	5.7	4.2	6.2
**5**	4.1	6.0	10.8	5.8	4.6	6.3
***s*.*e*.*m*.**	*0*.*04* **	*0*.*03* **	*0*.*12* *	*0*.*11*	*0*.*18*	*0*.*07*
*CP affected by P application*						
**Low**	4.0	5.9	10.0	5.8	4.2	6.0
**Medium**	4.4	5.8	10.7	5.8	4.6	6.3
**High**	4.0	5.9	10.9	6.0	4.7	6.3
***s*.*e*.*m*.**	*0*.*10* **	*0*.*09*	*0*.*17* **	*0*.*10*	*0*.*11* **	*0*.*05* **
*ADF affected by defoliation*						
**1**	35.3	27.3	17.2	29.6	30.2	27.9
**3**	38.0	29.3	20.2	34.0	33.8	30.5
**5**	34.7	26.5	12.5	29.2	31.7	26.9
***s*.*e*.*m*.**	*1*.*83*	*1*.*06*	*0*.*55* **	*1*.*36*	*1*.*44*	*0*.*72* *
*ADF affected by P application*						
**Low**	36.8	27.5	15.3	30.4	31.6	28.3
**Medium**	36.2	28.3	17.9	30.8	32.7	29.0
**High**	35.0	27.3	16.7	31.6	31.3	28.0
***s*.*e*.*m*.**	*0*.*60*	*0*.*65*	*0*.*53* *	*1*.*21*	*0*.*54*	*0*.*42*

**P<0.01;

*P<0.05.

There were significant (*P* < 0.01) differences in P concentration of *K*. *humilis*, *M*. *ruthenica*, forbs, dead matter and the mean between the P fertiliser treatments (Table 4). The P concentration in the herbage of all these species increased with increasing rates of P application. The P concentration of *E*. *nutans* followed the same trend although there was no significant difference between the treatments.

#### Crude protein

There were significant (*P* < 0.01 or 0.05) differences in the crude protein content of *E*. *nutans*, *K*. *humilis* and *M*. *ruthenica*, but not in the forbs, the dead matter or the mean of all species/categories, between the defoliation treatments (Table 4). The crude protein was generally higher in the herbage when pastures were cut to 3 cm (*E*. *nutans* and *K*. *humilis*) or 5 cm (*M*. *ruthenica*).

There were significant (*P* < 0.01) differences in the crude protein content of *E*. *nutans*, *M*. *ruthenica*, the dead matter and the mean, but not in *K*. *humilis* and forbs, between the P fertiliser treatments (Table 4). The crude protein of these species and the mean crude protein of all species were generally higher under the medium and/or high P treatments than the low P treatment.

**Fig 3:**
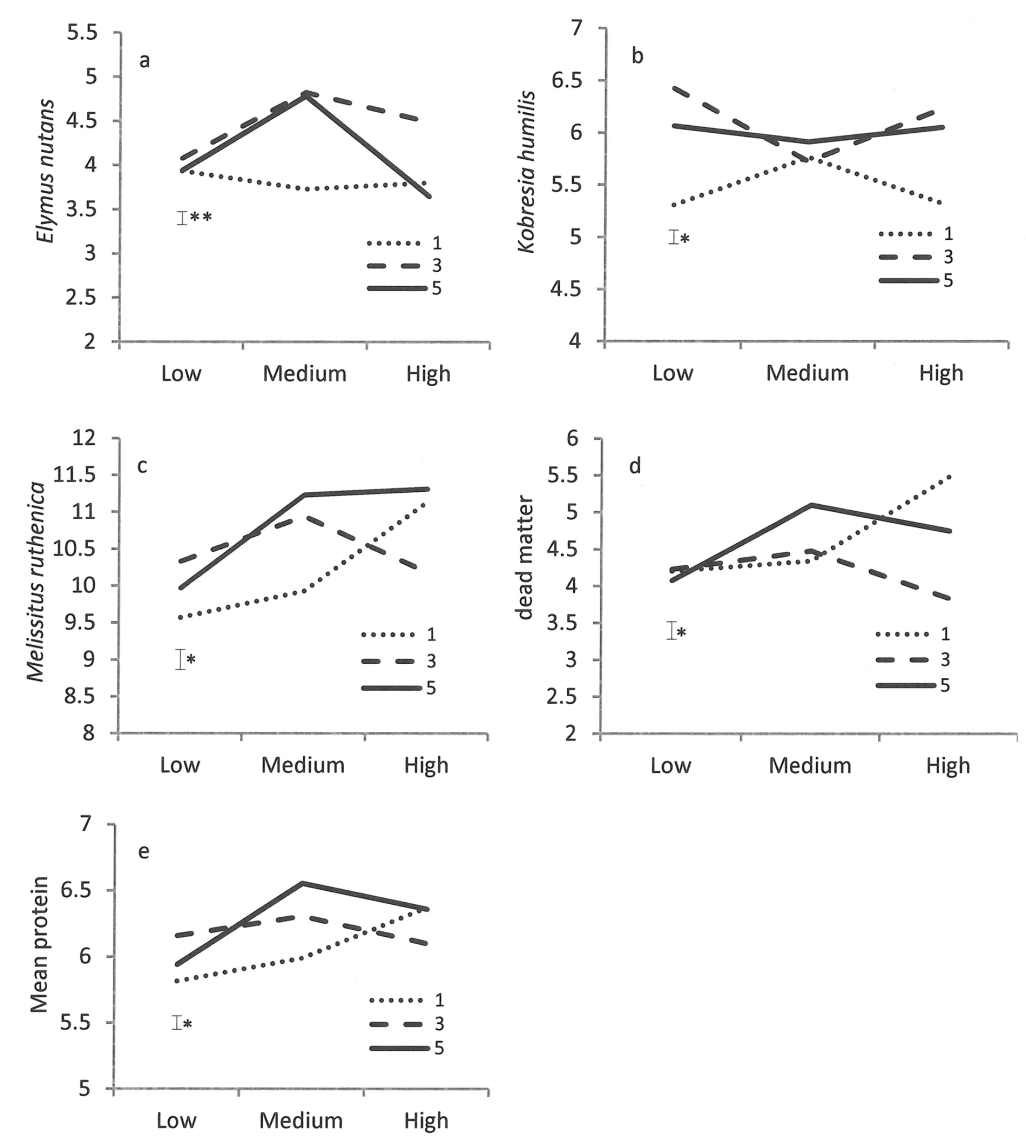
Interactions between defoliation regimes (cut to 1, 3 and 5 cm above ground) and fertiliser application (low, medium and high) on the protein content (%) of (a) *Elymus nutans*, (b) *Kobresia humilis*, (c) *Melissitus ruthenica* and (d) dead matter, and (e) the mean. Bars represent the standard error of the mean; **P < 0.01; *P < 0.05.

The crude protein content was generally higher in *M*. *ruthenica* (> 9.5%) than in other species (< 6.5%) and dead matter (< 5%). There were significant (*P* < 0.05 or 0.01) interactions in the crude protein content of *E*. *nutans*, *K*. *humilis* and *M*. *ruthenica*, dead matter or the mean between the defoliation treatments (Fig 3). For *E*. *nutans*, the crude protein content of the herbage increased from low P to medium P when the pastures were cut to 3 or 5 cm above ground, and declined from medium P to high P, particularly for the 5 cm defoliation treatment (Fig 3a). The crude protein content of the herbage remained unchanged regardless of P application when pastures were cut to 1 cm above ground. For *K*. *humilis*, the crude protein content of the herbage decreased from low P to medium P, then increased from medium P to high P when the pastures were cut to 3 or 5 cm above ground (Fig 3b). However, the crude protein content of the herbage for the 1 cm defoliation treatment changed in an opposite manner to the 3 or 5 cm defoliation treatments. For *M*. *ruthenica*, the crude protein content of the herbage increased from low P to medium P, then decreased or remained at similar level from medium P to high P when the pastures were cut to 3 or 5 cm above ground (Fig 3c). However, the crude protein content of the herbage for the 1 cm defoliation treatment increased with increasing P rates. The crude protein content of the herbage for the dead matter and the mean (Fig 3d and 3e) followed a similar trend as for *M*. *ruthenica*.

#### Acid detergent fibre

There were significant (*P* < 0.01 or 0.05) differences in the ADF content of *E*. *nutans* and the mean across all species between the defoliation treatments (Table 4). The ADF was higher in the herbage of *E*. *nutans* and all pasture species when pastures were cut to 3 cm above ground. There was no significant difference in the ADF content of *K*. *humilis*, *M*. *ruthenica*, forbs and the dead matter.

There was a significant (*P* < 0.05) difference in the ADF content of *M*. *ruthenica* between the P fertiliser treatments (Table 4). The ADF of *M*. *ruthenica* was 17.9% in the medium P treatment, higher than the low P treatment. There were no difference in the ADF content of all other species/categories or the mean.

### Soil moisture and nutrients

#### Gravimetric soil moisture

There were significant (*P* < 0.05 or 0.01) differences in gravimetric soil moisture between the P fertiliser treatments and between soil depths (Table 5). The soil moisture under the low P treatment was 18.9%, 8–10% higher than the medium and high P treatments. The soil moisture at the top soil (0–10 cm) was 22.0%, 33–46% higher than that in the 10–20 cm and 20–30 cm soil depths. There was no significant difference in soil moisture between the defoliation treatments.

**Table 5:** Mean gravimetric soil moisture (SM; %) of different soil depths (cm) under various defoliation regimes (cut to 1, 3 and 5 cm above ground) and P application (low, medium and high).

Defoliation	SM	Phosphorus	SM	Depth	SM
**1**	17.9	Low	18.9	0–10	22.0
**3**	19.0	Medium	17.5	10–20	16.5
**5**	16.7	High	17.2	20–30	15.1
***s*.*e*.*m*.**	*0*.*65*	*s*.*e*.*m*.	*0*.*49* *	*s*.*e*.*m*.	*0*.*49* **

**P<0.01;

*P<0.05.

#### Soil nutrients

There was a significant (*P* < 0.01) difference in total soil P concentration between the defoliation treatment (Table 6). The mean total P concentration was 216.8 mg/kg soil when pastures were cut to 1–3 cm above ground, 5% higher than that cut to 5 cm above ground. There were no significant differences in available P, available K, total K, available N and total N between the defoliation treatments.

**Table 6:** Soil nutrient (P_e_—available phosphorus; P_t_—total phosphorus; K_e_—available potassium; K_t_—total potassium; N_e_—available nitrogen; N_t_—total nitrogen) concentrations (mg/kg soil) under various defoliation regimes (cut to 1, 3 and 5 cm above ground) and phosphorus application (low, medium and high).

Defoliation	P_a_	P_t_	K_a_	K_t_	N_a_	N_t_
*Defoliation*						
**1**	1.4	216.9	134.8	412.2	293.9	376.3
**3**	1.4	216.7	138.2	390.0	286.8	384.5
**5**	1.2	205.5	138.2	399.0	272.1	358.3
***s*.*e*.*m*.**	*0*.*04*	*1*.*12* **	*4*.*34*	*9*.*10*	*5*.*40*	*7*.*61*
*Phosphorus*						
**Low**	1.2	207.9	137.0	424.7	275.9	364.4
**Medium**	1.4	214.3	140.6	362.7	287.1	381.6
**High**	1.4	216.9	133.6	413.8	289.8	373.2
***s*.*e*.*m*.**	*0*.*06*	*2*.*70*	*4*.*17*	*11*.*51* **	*4*.*37*	*9*.*84*

**P<0.01.

There was a significant (*P* < 0.01) difference in total soil K concentration between the P fertiliser treatment (Table 6). The total K concentration for the medium P treatment was 362.7 mg/kg soil, 12–15% lower than the low or high P treatments. There were no significant differences in available P, total P, available K, available N and total N between P fertiliser treatments.

**Fig 4:**
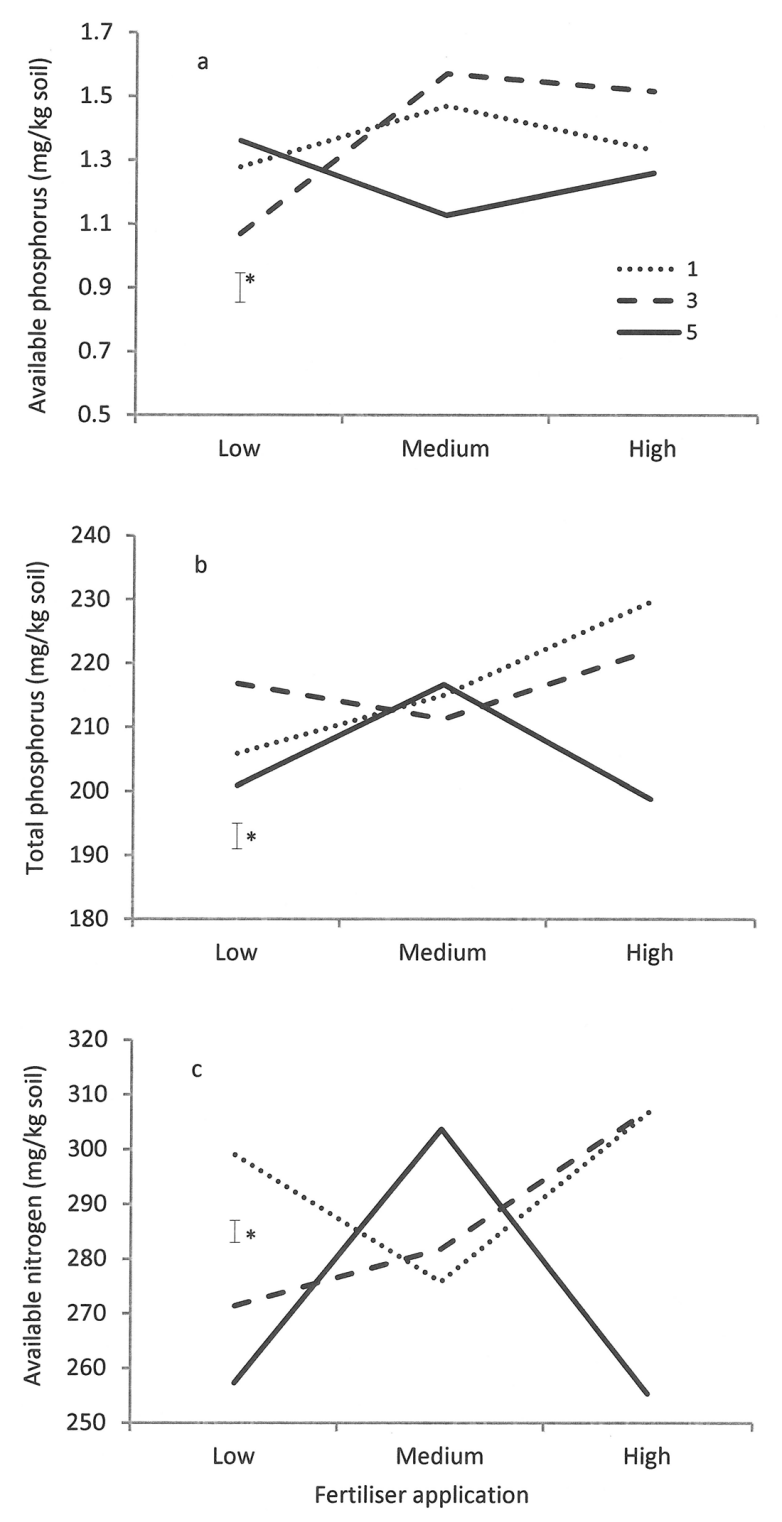
Interactions between defoliation regimes (cut to 1, 3 and 5 cm above ground) and phosphorus application (low, medium and high) on (a) effective and (b) total phosphorus and (c) effective nitrogen in the soil. Bars represent the standard error of the mean; *P < 0.05.

There were significant (*P* < 0.05) interactions in available and total P and available N between the defoliation and P fertiliser treatments (Fig 4). The available P under 1 cm and 3 cm cutting treatments increased from Low P to medium P then declined from medium P to High P treatment; however, the available P under 5 cm cutting treatment declined from Low P to medium P then increased from medium P to High P treatment (Fig 4a). The total P under 3 cm cutting treatment declined from low P to Medium P treatment whereas that under 1 cm or 5 cm cutting treatment increased from low P to Medium P (Fig 4b). The total P under 1 cm and 3 cm cutting treatments further increased from medium P to high P whereas that under 5 cm cutting treatment declined sharply from Medium P to high P.

The total N under 3 cm cutting treatment increased with P application (Fig 4b). The total N changed in a completely opposite manner between the 1 cm and 5 cm cutting treatments, i.e. total N under 1 cm cutting was lowest at the medium P treatment whereas that under 5 cm cutting peaked at the medium P treatment (Fig 4c).

## Discussion

### Plant growth and competition

The study has revealed that the total herbage yield of the alpine pasture in the Qinghai-Tibet Plateau was significantly increased by P application, and to a lesser degree by more intensive defoliation. This was in line with the findings of Yang et al. [15] and Wang et al. [28] that fertiliser application could play a more important role in improving pasture growth in Qinghai-Tibet Plateau. In their studies, the fertiliser used was DAP which contains 18% N and 20% P; therefore, it’s difficult to differentiate the effects between N and P. Given grasses were the dominant species in these studies and fertiliser application at higher rates was found to have a detrimental impact on the growth and competition of legumes and sedge [15], N may have had a greater effect than P under higher fertiliser rates in these studies due to stronger responses to N by grasses that competed against the legumes and sedge.

In this study, application of P fertiliser has clearly promoted the growth and competition of *M*. *ruthenica*, a predominant species among the legumes in the sward. Prior to the P treatment, the botanical composition of *M*. *ruthenica* was only 18%. This was increased to 25–30% in year 2 and 3 after P fertiliser treatment. This was translated to an increase in herbage production from 375 kg DM/ha in year 1 to 875 kg DM/ha in year 3, an increase by 133%. A number of studies [20,21] have reported that P addition to pastures could improve the growth of legumes. An increase of legumes in the herbage component of a pasture may not only mean more DM production, but also better pasture nutritive value, more N fixed in the grazing systems and less N leaching and nitrous oxide emissions compared with N fertiliser application [29,30].

Defoliation intensity interacted with P application on herbage yield of *M*. *ruthenica*, probably due to competition of this species with other plants in the sward. The herbage yield of this species increased from low P to medium P regardless of defoliation intensity, but declined from medium P to high P when pastures were cut to 5 cm above ground, reflecting stronger competition from grasses under high P and high cutting residual (5 cm) treatments. *M*. *ruthenica* was a short plant (mean height = 13.3 cm), similar in height to *K*. *humilis* (mean height = 13.6 cm), but much shorter than *E*. *nutans* (mean height = 60.5 cm). At low defoliation intensity (or high cutting residual), *M*. *ruthenica* could have been disadvantaged in competition with taller companion species due to shading by these plants. Martin and Chambers reported that close defoliation can alter the competitive relations between species in the sward through increased light intensity reaching the soil surface and the remaining vegetation, and through increased water or nutrient availability [31]. The plant frequency of *M*. *ruthenica* was highest at medium P application and medium defoliation intensity (3 cm above ground), indicating that these were probably optimum grazing and P fertiliser management levels to achieve both high production and sward stability (persistence) by this species.

Similar response was found for *K*. *humilis*, a dwarf plant, which had lowest herbage yield (371 kg DM/ha) when pastures were cut to 5 cm above ground. *K*. *humilis* is a highly palatable and nutritious sedge that is very tolerant to grazing and cold winter of alpine meadow [32]. An interaction between fertiliser and defoliation was also found to affect the regrowth of *K*. *humilis* by Zhang et al. [33].

Unlike *K*. *humilis* and *M*. *ruthenica*, *E*. *nutans* did not respond significantly either to defoliation or fertiliser treatment although the species appeared to produce poorly when pastures were cut to 1 cm above ground and addition of P fertiliser seemed to have promoted its growth. Gu et al. compared *E*. *nutans*, *Festuca sinensis* and *Festuca ovina* in eastern Qinghai-Tibet plateau and found that there was little difference in the relative competitiveness between these species when the plants were not clipped, cut to 6 cm above ground or cut to 2 cm above ground [23]. *E*. *nutans* is a tall and erect grass that may not tolerate close defoliation well as the short and prostrate species. Therefore, laxer grazing may have given the plants opportunities to recover and recharge for regrowth.

The decline in the botanical composition of forbs from year 1 (>60%) to years 2 and 3 (<40%) was probably attributed to overall stranger growth and competition of the grass, legume and sedge species in the sward over time with P application and defoliation treatments for most of the plots.

### Pasture nutrients

The P concentration of all plant components increased with increasing P application except *E*. *nutans* that showed a similar trend but did not differ significantly between P treatments, reflecting that P uptake by plants was effective although there were differences between species in the current study. The plant-available P concentration on the study site was <1.5 mg/kg soil, which was far lower than the optimum P level for crops (>20 mg/kg soil) in China [19]. Soils that are P-deficient and consequently cannot support optimum production are often improved by applying P fertiliser and/or manure to promote plant growth [17]. Insignificant response of *E*. *nutans* to P application was probably attributed to its relatively lower critical P requirement than other species. A number of studies [34–37] reported that most temperate grasses are more effective at obtaining P from soil than their companion legume species and have lower critical P requirements.

As for herbage yield and plant frequency, the P concentration of *M*. *ruthenica* was highest when pastures were cut to 3 cm above ground probably due to stronger competition of this species at this defoliation intensity. However, the P concentration of *E*. *nutans* was highest when pastures were cut to 1 cm above ground, a defoliation intensity level that was detrimental to its growth and persistence (plant frequency). It is not clear why *E*. *nutans* increases its P absorption under more intense defoliation. In part, it may have been attributed to its responses to defoliation stress by increasing P uptake to compensate growth and retain competitive ability in the sward. Gu et al. found that *E*. *nutans* is a highly competitive species when sown in mixture with *Festuca* spp. [23].

The interactions in protein content of various pasture components between defoliation and P fertiliser were complicated and protein content was generally higher at medium P rate and a defoliation intensity of 3–5 cm above ground. The medium P rate appeared to be the optimum rate for plant growth and protein content of most species in this study, and further increase in P rate did not have much added value. Given the P concentration of the soil was very low and application at the high rate (40 kg P/ha) in this study was still much lower than the recommended soil P (61 kg P/ha) for soils at this P level [19], there may have been other factors such as other soil elements that limited pasture performance. Overall, the protein content was low and fibre content high due to the short growing season and pastures being mature at their harvest. However, the protein content of *M*. *ruthenica* was much higher and fibre content much lower than other species/components, demonstrating the importance of this species in improving the nutritive value of the alpine pastures.

### Soil moisture and nutrients

The gravimetric soil moisture in August 2013 decreased with increasing soil depths, reflecting typical soil moisture distribution in wet seasons. The soil moisture also decreased with increasing P fertiliser rates, probably due to improved pasture growth that had used more water from the 0–30 cm soil profile. However this trend may change in longer term since more active growth of roots as a result of more P addition would create a larger buffer through root-created pores and increase soil water retention [38,39].

There are numerous complex interactions between defoliation/fertiliser management and soil nutrient dynamics. Responses of soil nutrients to defoliation and fertiliser application primarily depend on the type of fertiliser applied, the pasture plants that use the nutrients, defoliation that alters plant growth, soil chemical reactions and soil physical conditions. In general, soil available and total P concentration increased from low P to medium/high P treatments when pastures were cut to 1–3 cm above ground. However, when pastures were cut to 5 cm above ground, the available P was lowest but total P highest at the medium P treatment. The low available P concentration at the medium P and low defoliation treatment was probably related to higher P utilisation for the treatment due to enhanced plant growth. The total K concentration had a similar response, being lowest for the medium P treatment. It is worthwhile noting that there could be a considerable amount of P in the stubble that was left over after harvest and was not measured, which may help explain why the total P concentration in the soil at 5 cm cutting intensity was lower than the other defoliation treatments.

The sodium level in the study was very high (>270 mg/kg soil), which may indicate high soil sodicity of the site. Sodic soils are more commonly found in areas of low rainfall since there is less opportunity for salts to leach through the soil profile [40]. Given the annual rainfall in Qinghai-Tibet Plateau is low and soil pH is high (>7) in many regions, soil sodicity needs more attention for future research. High sodicity not only reduces the quantity of available P and K in the soil [41], but also weakens the aggregates in the soil, causing structural collapse and closing-off of soil pores.

### Practical implications

Much of the native grassland in Qinghai-Tibet Plateau is overexploited and the soil fertility is depleted. Application of various fertilisers has been demonstrated to considerably improve the alpine grassland production, and N and P were the major nutrients that restrict grass growth in the alpine meadow [42–44]. Although the Chinese government has invested a great deal to improve the productivity and groundcover of the alpine pasture in Qinghai-Tibet Plateau over the past decades, it is necessary to develop long-term strategies combining the profitability and sustainability for local farming communities.

A critical finding of this study is that the herbage yield of *M*. *ruthenica* was increased by 68% and total pasture yield by 25%, simply by applying 20 kg P/ha to the pasture. This equates to an increase of approximately 1 sheep unit/ha based on daily intake of livestock being 1.5 kg DM/sheep. In addition, the N fixed by the increased legume content (317 kg DM/ha) was about 19.8 kg N/ha, based on average N fixed by legumes (e.g. *M*. *ruthenica*) in an alpine *Kobresia* grassland of the Tibet Plateau [45], a rate that is hard to achieve with N fertiliser application for low-input native pasture grazing systems. Nitrogen fixed by legumes in grassland systems not only saves the cost of N fertiliser inputs, but also reduces N losses through leaching and nitrous oxide emissions associated with applied N fertilisers [29]. Therefore, application of P fertiliser to promote legume growth and N fixation in the native grassland of the Qinghai-Tibet Plateau would be a more economical, productive and sustainable practice than application of mixed N and P fertilisers that can suppress legume growth at higher rates [15]. Apart from P addition to promote legumes in the plant community, it is also important to explore plant species with the ability to acquire soil P such as species with cluster roots [46] and some legume crops that can mobilise soil-bound phosphorus [47] in these P-impoverished soils. This deserves further investigation.

Since grazing is a key element of the grassland ecosystems [6,48], appropriate grazing management is critical for maintaining grassland productivity and sustainability. Traditionally, the grazing management in Qinghai-Tibet Plateau is nomadic, which is driven by temperature and seasons. Over the past decades, considerable research and investment have been made on grazing management (e.g. rest of pastures and grazing ban) to improve the pasture cover and yield in Qinghai-Tibet Plateau; however, there has often been conflicts in grazing management to balance the demand for increased productivity by the local herders and over-exploitation of grassland [22].

Although pasture rest and grazing ban could be an effective tool to rehabilitate badly degraded pastures, grazing at an appropriate intensity together with optimum whole farm management systems is ultimately a long-term solution to achieve both improved productivity and groundcover. Since native pastures are usually diverse in species composition and individual species generally require different defoliation intensities to grow and persist at their best, it is difficult to work out one grazing policy that suits all species in the community. For instance, *E*. *nutans* is a taller grass and requires laxer grazing to achieve better yield and persistence in this study, whereas *M*. *ruthenica* and *K*. *humilis* are shorter and closer defoliation could prevent these species being choked by taller species. Interestingly, defoliation at 3 cm above ground appeared to be an ideal compromise for all major species in the current study, giving beneficial outcomes in terms of plant frequency (or groundcover), herbage yield, herbage nutritive value, soil moisture and soil nutrients. In practice, this defoliation pressure, when used together with medium P fertiliser application, has the potential to deliver long-term production and sustainability benefits for similar pasture types in Qinghai-Tibet Plateau. It should be noted that the results from this study were derived by cutting and further studies with grazing by livestock are necessary to confirm the findings. In addition, since the study was undertaken on one site only, it is necessary to conduct research for a wider range of pasture types in the Plateau. While harsh grazing management policies that have pros and cons have been implemented for decades, the concept of protecting the grassland by properly utilising the pastures need to be incorporated in pasture grazing management regulations as well as communicated to local herders and their advisors.

## Supporting Information

S1 Table The source of variation and degree of freedom (d.f.) for split-plot model, split-plot model with repeated measures and split-split-plot model with repeated measures used to analyse the data of this study. Rep—replicate; Cut—cutting treatment; Fert—fertiliser treatment; Dates—dates for repeated measures; Depth—soil depth.
(DOCX)
