# Supplementary material for: Phosphorus and Defoliation Interact and Improve the Growth and Composition of the Plant Community and Soil Properties in an Alpine Pasture of Qinghai-Tibet Plateau
Source: PLoS One. 2015 Oct 29;10(10):e0141701. doi: 10.1371/journal.pone.0141701 (PMC4626130; doi:10.1371/journal.pone.0141701)
Supplement: S1 Table — Rep—replicate; Cut—cutting treatment; Fert—fertiliser treatment; Dates—dates for repeated measures; Depth—soil depth. (DOCX) [file pone.0141701.s001.docx]

**The source of variation and degree of freedom (d.f.) for split-plot model, split-plot model with repeated measures and split-split-plot model with repeated measures used to analyse the data of this study.** Rep – replicate; Cut – cutting treatment; Fert – fertiliser treatment; Dates – dates for repeated measures; Depth – soil depth.

| Split-plot model |  | Split-plot model with repeated measures |  | Split-split-plot model with repeated measures |  |
| --- | --- | --- | --- | --- | --- |
| Source of variation | d.f. | Source of variation | d.f. | Source of variation | d.f. |
| Rep | 3 | Rep | 3 | Rep | 3 |
| Rep.Cut stratum |  | Rep.Cut stratum |  | Rep.Cut stratum |  |
| Cut | 2 | Cut | 2 | Cut | 2 |
| Residual | 6 | Residual | 6 | Residual | 6 |
| Rep.Cut.Fert stratum |  | Rep.Cut.Fert stratum |  | Rep.Cut.Fert stratum |  |
| Fert | 2 | Fert | 2 | Fert | 2 |
| Cut.Fert | 4 | Cut.Fert | 4 | Cut.Fert | 4 |
| Residual | 18 | Residual | 18 | Residual | 18 |
| Total | 35 | Rep.Cut.Fert.Dates stratum | | Rep.Cut.Fert.Depth stratum | |
|  |  | Dates | 2 | Depth | 2 |
|  |  | Cut.Dates | 4 | Cut.Depth | 4 |
|  |  | Fert.Dates | 4 | Fert.Depth | 4 |
|  |  | Cut.Fert.Dates | 8 | Cut.Fert.Depth | 8 |
|  |  | Residual | 54 | Residual | 54 |
|  |  | Total | 107 | Rep.Cut.Fert.Depth.Dates stratum | |
|  |  |  |  | Date | 1 |
|  |  |  |  | Cut.Date | 2 |
|  |  |  |  | Fert.Date | 2 |
|  |  |  |  | Depth.Date | 2 |
|  |  |  |  | Cut.Fert.Date | 4 |
|  |  |  |  | Cut.Depth.Date | 4 |
|  |  |  |  | Fert.Depth.Date | 4 |
|  |  |  |  | Cut.Fert.Depth.Date | 8 |
|  |  |  |  | Residual | 81 |
|  |  |  |  | Total | 215 |
